# Supplementary material for: The miR-182-5p/NDRG1 Axis Controls Endometrial Receptivity through the NF-κB/ZEB1/E-Cadherin Pathway
Source: Int J Mol Sci. 2022 Oct 14;23(20):12303. doi: 10.3390/ijms232012303 (PMC9602861; doi:10.3390/ijms232012303)
Supplement: Supplementary file 1 [file ijms-23-12303-s001.zip › Supplementary materials.pdf]

Supplementary Table S1. Primer sequences for qRT-PCR (quantitative reverse transcription-polymer chain reaction).

Gene (Accession numbers)	Primer sequences	Annealing temperature	Size of amplicon (bp)
NDRG1 (NM_001135242)	Forward: 5′- taccgccagcacattgtgaa -3′<br>Reverse: 5′- gccacagtccgccatctt-3′	56°C	240
P65 (NM_001145138)	Forward: 5′- tgcagaaagaggacattgaggt-3′<br>Reverse: 5′- ctgcatggagacacgcacaggag -3′	56°C	158
ZEB1 (NM_001174096)	Forward: 5′- aagaattcacagtggagagaagcc -3′<br>Reverse: 5′- cgtttcttgcagtttgggcat -3′	56°C	51
CDH1 (NM_004360)	Forward: 5′-tcagcgtgtgtgactgtgaa -3′<br>Reverse: 5′-cctccaagaatccccagaat -3′	56°C	100
GAPDH (NM_002046)	Forward: 5′- acagtcagccgcatcttctt -3′<br>Reverse: 5′- acgaccaaatccgttgactc -3′	56°C	94

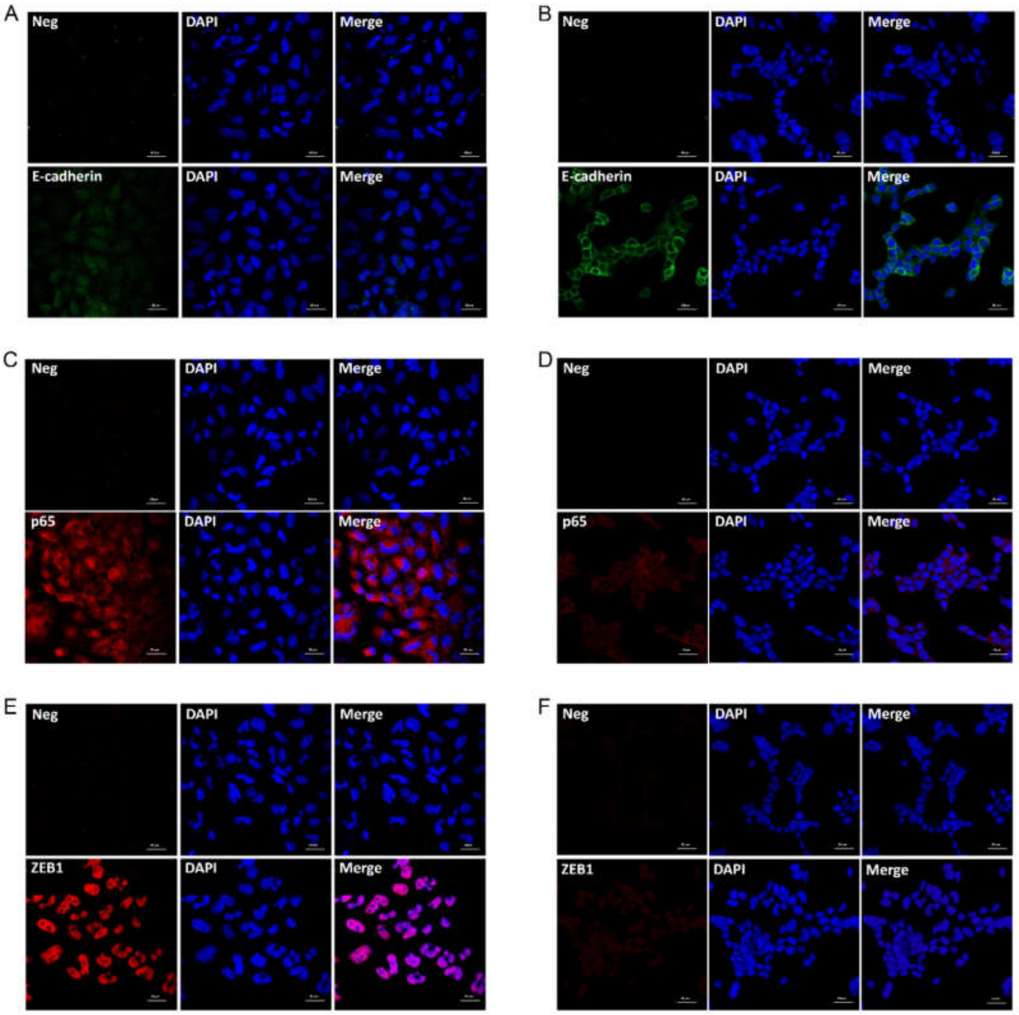

Supplementary Figure S1. Immunofluorescence-staining of E-cadherin, p65, ZEB1 and IgG negative controls. Images of E-cadherin and IgG expression in AN3-CA cells (A) and RL95-2 cells (B). Images of p65 and IgG expression in AN3-CA cells (C) and RL95-2 cells (D). Images of ZEB1 and IgG expression in AN3-CA cells (E) and RL95-2 cells (F). All images are shown at 400X magnification. Scale bars indicate 20 µm. Nuclei were stained with 4′,6′-diamidino-2-phenylindole dihydrochloride (DAPI).
